# Cyclin-Dependent Kinase 5 Contributes to Bruton’s Tyrosine Kinase Inhibitor Resistance via the IRE1α/XBP1 Axis in Mantle Cell Lymphoma

**DOI:** 10.21203/rs.3.rs-9500406/v1

**Published:** 2026-05-08

**Authors:** Alexey Danilov, Sonia Rodriguez-Rodriguez, Dan Vuong, Huijia Yan, Andrew Chen, Carly Roleder, Tiana huynh, Vi Lam, Haifeng Shen, Jibin Zhang, Xinzhou Ge, Lucy Ghoda, Stacy Pak, Vu Ngo, Geoffrey Shouse, Tycel Phillips, Lili Wang

**Affiliations:** City of Hope; City of Hope; City of Hope; City of Hope; City of Hope; City of Hope; City of Hope; Oregon Health and Science University; City of Hope; City of Hope National Medical Center; Oregon State University; Department of Hematology and HCT, City of Hope; City of Hope; Beckman Research Institute, City of Hope; City of Hope National Medical Center; City of Hope; Columbia University Irvine Medical Center

## Abstract

The mechanisms of resistance to Bruton tyrosine kinase inhibitors (BTKi) in MCL are not well understood. We found that cyclin-dependent kinase 5 (CDK5), a serine/threonine kinase, was upregulated in ibrutinib-resistant MCL cell lines and in primary MCL cells obtained from patients who progressed on BTKi. Furthermore, primary MCL cells upregulated CDK5 in BAFF/CD40L stromal conditions, and *CDK5* mRNA overexpression in primary MCL tumors was associated with inferior outcomes. Genetic and pharmacologic (GFB-12811) manipulation of CDK5 in MCL cell lines revealed that CDK5 contributed to proliferation and ibrutinib resistance *in vitro*, while engineered expression of CDK5 in JeKo-1 cells shortened survival in a murine xenograft model. CDK5 was upregulated by B-cell receptor and BAFF signaling and complexed with BTK. Mass spectrometry analysis of CDK5-manipulated cells revealed that cell cycle and metabolism-related pathways were most affected by CDK5; 29 kinases were differentially active, and Src kinase activity correlated with CDK5 expression. CDK5 complexed with IRE1α and Xbp1, promoted IRE1α phosphorylation and increased Xbp1 levels thus facilitating unfolded protein response. Meanwhile, Xbp1 knockdown resensitized MCL cells to ibrutinib. Collectively, CDK5 promotes BTKi resistance via modulation of the XBP1/IRE1α axis of the UPR pathway and represents a potential therapeutic target in MCL.

## INTRODUCTION

Mantle cell lymphoma (MCL) is a rare and aggressive subtype of B-cell non-Hodgkin lymphoma (NHL) that accounts for ~ 6% of all NHL cases. It is defined by the chromosomal translocation t(11:14),^1^ and frequent mutations in ATM, while high-risk patients carry additional genetic abnormalities such as *TP53* mutations and complex karyotype.^2^ MCL is also characterized by increased B-cell receptor (BCR) signaling. Bruton tyrosine kinase inhibitors (BTKi) have transformed the treatment paradigm in MCL patients.^3–5^ However, all patients ultimately develop resistance, with prognosis particularly poor among patients who exhibit resistance to BTKi.^6^

Recent efforts have been directed to understand drug resistance mechanisms in MCL, particularly in a setting of progression on BTKi’s. In chronic lymphocytic leukemia (CLL), acquired mutations in *BTK* (e.g. C481S) and phospholipase Cγ2 (*PLCG2*), account for the majority of cases of resistance to BTKi, but they are not as common in MCL.^7^ Alternative mechanisms, such as constitutive NF-κB activation, direct tumor microenvironment interaction or *CCND1* mutations have been implicated in BTKi resistance,^8–11^ providing justification for exploration of therapeutic targets outside the BTK signaling pathway.

CDKs comprise a family of serine-threonine kinases that are implicated in fundamental biological processes such as cell cycle progression, transcriptional regulation or homologous recombination (HR)-mediated DNA repair and are key players for cancer development and treatment resistance. CDK5 is unique because although it shares high homology with other CDK members^12^ it is not activated by binding to cyclins. Instead, CDK5 complexes with the specific activation subunits p35 and p39, and their cleavage products, p25 and p29.^13, 14^ CDK5 was first discovered for its crucial role in neural development, differentiation and migration.^13^ In recent years, several studies have highlighted its oncogenic potential in solid tumors.^15, 16^

In this study we show that CDK5 is overexpressed in MCL cells which developed acquired resistance to BTKi, and that pharmacological or genetic blockade of CDK5 partially abrogates ibrutinib resistance. Taken together, our data supports CDK5 as a novel therapeutic target in MCL.

## MATERIALS and METHODS

### Cell lines and patient samples

JeKo-1, Mino, Maver-1, Z138, Granta 519, HEK293T cell lines were obtained from American Type Culture Collection (ATCC) and German Collection of Microorganisms and Cell Cultures (DSMZ). Peripheral blood from MCL specimens were acquired from patients following approval by the Institutional Review Board and after written consent at the City of Hope National Medical Center (Duarte, CA).

### Cell culture and reagents

Cells were cultured in RPMI-1640 medium supplemented with 15% fetal bovine serum, 100 U/mL penicillin, 100 μg/mL streptomycin, 2 mM l-glutamine, 25 mM HEPES, 100 μM non-essential amino acids, and 1 mM sodium pyruvate.

GFB-12811 and A419259 were sourced from MedChemExpress LLC. Ibrutinib, Dasatinib, Venetoclax, Bortezomib and Cycloheximide were sourced from Selleck Chemicals LLC.

### Statistical analysis

At least 3 biological replicates were used in all experiments shown throughout the manuscript, unless noted otherwise. Error bars represent standard error of the mean (SEM) unless otherwise indicated. Statistical analysis was performed with Student t test or one-way ANOVA with Tukey’s post-hoc test, when indicated. For survival, Kaplan-Meier analysis with Logrank (Mantel-Cox) was used. All analysis was performed in GraphPad Prism software. *p < 0.05, **p < 0.01 and ***p < 0.001 throughout the manuscript.

## RESULTS

### Ibrutinib resistance is associated with a distinct transcriptional program in MCL cells

**Figure 1.**
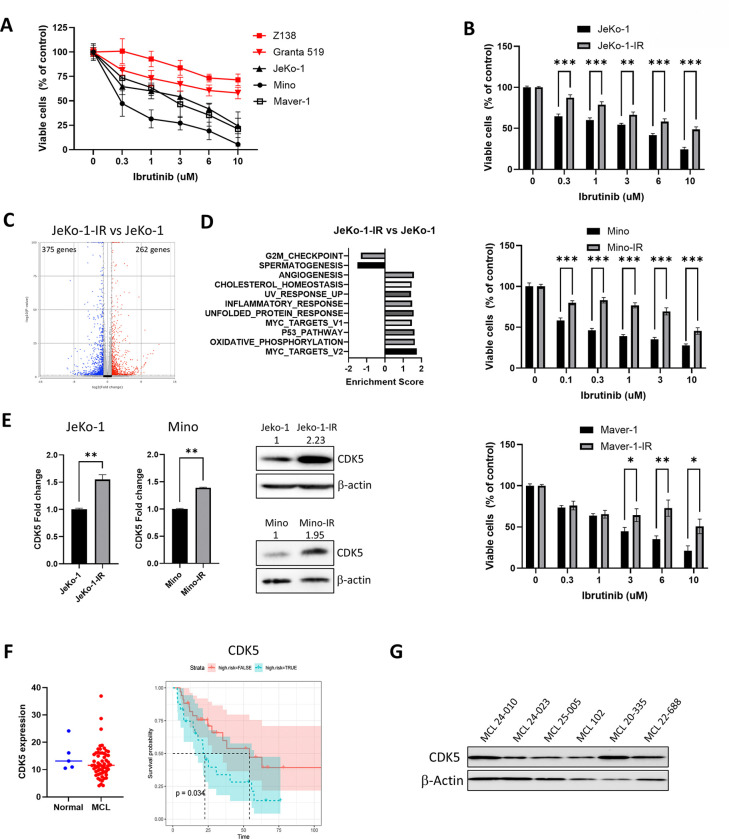
Ibrutinib-resistant MCL cells overexpress CDK5 (A) MCL cell lines were treated with the indicated doses of ibrutinib or vehicle control for 72 hours. Cell proliferation was measured by colorimetric MTS assay. Resistant (red) and sensitive (black) cell lines are shown (n=9 in 3 independent experiments here and thereafter). (B) Proliferation of ibrutinib-resistant vs. parental cell lines in the presence of ibrutinib (n=9). (C) Parental and IR Jeko-1 cells were subjected to bulk RNA-Seq. Volcano plots show differential gene expression, upregulated (red) and downregulated (blue), in JeKo-IR cells (n=3) Padj<0.05. (D) GSEA Pathway analysis of JeKo-IR cells vs. parental showing most significantly enriched pathways ranked by NES (normalized enrichment score) (n=3). (E) *CDK5* mRNA and protein expression by RT-PCR in IR and parental cells (n=3–5). Representative immunoblot shown. Values above each lane represent the mean relative expression (densitometry) normalized to β-actin from three independent experiments. (F) CDK5 mRNA expression in MCL patients compared to normal individuals (top) and Kaplan-Meyer survival curves of MCL patients grouped based on high (red) or low (green) CDK5 expression (bottom). Long-rank test *p<0.05. (G) CDK5 protein levels in MCL cells obtained from patients who progressed on BTKi. Data are mean ± SEM, *p<0.05, **p<0.01 and ***p<0.001.

To begin understanding resistance to BTKi, we evaluated sensitivity of MCL cell lines to ibrutinib. Of the 5 tested MCL cell lines, Z138 and GRANTA-519 exhibited strong primary resistance to ibrutinib with over 50% of the cells surviving after treatment with 10 μM of the drug (Figure 1A). Meanwhile, JeKo-1, Maver-1 and Mino cells were susceptible to ibrutinib with IC_50_ of 4.7±1.5, 2.7±1 and 0.25±0.14 μM, respectively. The latter cell lines were chosen to generate acquired BTKi resistance. To this end, cells were cultured for up to 6 months in increasing concentrations of ibrutinib (up to 10 μM). The resultant cell lines, hereafter referred to as JeKo-1-IR, Maver-1-IR and Mino-IR, exhibited resistance to ibrutinib relative to parental cells with >50% cells alive in 10 μM drug concentration (Figure 1B). While parental JeKo-1 and Mino cell lines manifested apoptosis upon treatment with ibrutinib, IR-cells were not susceptible to the drug (Supplemental Figure 1A).

We next conducted RNA-Seq analysis of JeKo-1 parental and JeKo-1-IR cells to identify transcriptional differences brought on by acquired ibrutinib resistance. Expression of 14,003 protein-coding genes was detected in JeKo-1-IR cells. Using Partek Flow analysis and a cutoff of at least 2-fold change, we identified 262 upregulated and 375 downregulated genes in JeKo-1-IR compared to JeKo-1 cells (padj<0.05; Figure 1C and Supplemental Figure 1B). Gene Set Enrichment Analysis (GSEA) identified ‘G2M Checkpoint’ as the key pathway that was most significantly associated with the downregulated genes in IR cells, while ‘Unfolded Protein Response’ (UPR), ‘Inflammatory Response’, ‘Myc targets’, ‘oxidative phosphorylation’, and ‘p53 pathway’ were significantly upregulated (Figure 1D; Supplemental Figure 1C). Somatic mutational analysis of our RNA-seq data revealed 37 and 28 mutations in JeKo-1-IR and Mino-IR respectively, with majority of them being transcription factors, scaffolding proteins and mitochondrial proteins involved in immune regulation, cell proliferation and apoptosis (Supplemental Tables 2–3).

Furthermore, we identified cyclin-dependent kinase-5 (CDK5) as a relevant candidate gene with a fold change of 2.15 (p=2.64^−110^; Supplemental Figure 1D). Using RT-PCT and immunoblotting, we confirmed that CDK5 mRNA and protein levels were upregulated in Jeko-IR and Mino-IR cell lines compared with parental cell lines (Figure 1E). We detected expression and binding of the known CDK5 partner p35 in MCL cells^13, 14^ (Supplemental Figure 1E). Given the emergent role of CDK5 in cancer we sought to further investigate its effects in MCL BTKi resistance.

Here we conducted an analysis of RNA-seq data from a cohort of treatment-naive MCL patients versus normal B cells (n=5 and 58, respectively).^17^ CDK5 was expressed in all cases and mRNA levels were comparable to normal B cells (Figure 1F). However, we observed significant variability in CDK5 mRNA levels in MCL. Using mean expression level as a dichotomous cutoff, we found that higher CDK5 mRNA expression was associated with inferior survival in this cohort (Figure 1F). Finally, we analyzed circulating MCL cells isolated from PBMCs from patients with MCL who had developed resistance to a BTKi (ibrutinib, acalabrutinib) and observed high CDK5 protein expression in these patients (Figure 1G).

Thus, CDK5 overexpression was associated with inferior outcomes in MCL and with resistance to BTKi.

### CDK5 contributes to ibrutinib resistance

**Figure 2.**
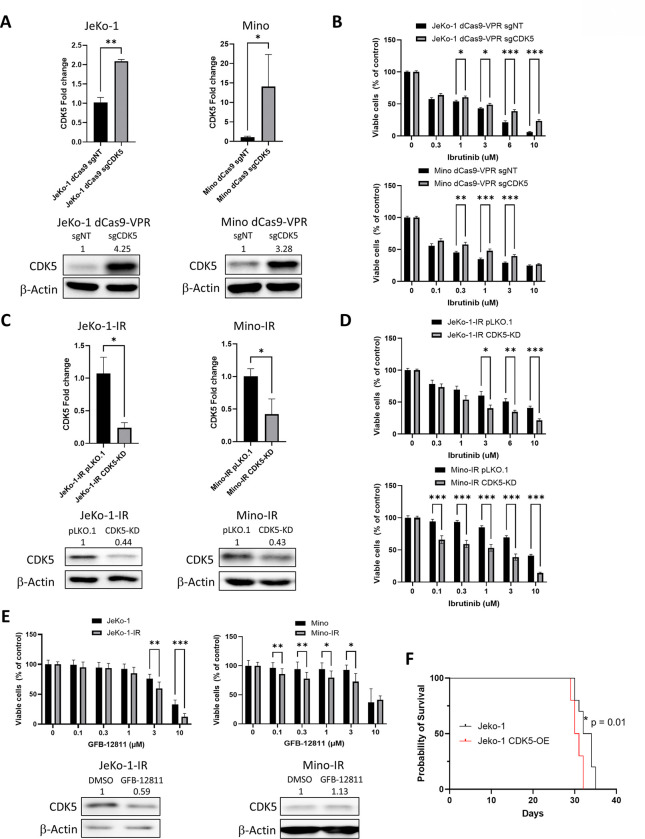
CDK5 is integral to Ibrutinib Resistance in Acquired Ibrutinib Resistance models (A) JeKo-1 and Mino cell lines were transduced with CDK5 or vector control were analyzed for *CDK5* mRNA levels by RT-PCR and protein levels by immunoblotting (n=3). (B) CDK5-overexpressing and control cells were treated with ibrutinib or vehicle control for 72 hours; proliferation was measured by colorimetric MTS assay (n=9). (C) JeKo-1-IR and Mino-IR cell lines were transduced with sh-CDK5 or vector control and analyzed for *CDK5* mRNA levels by RT-PCR and CDK5 protein levels by immunoblotting (n=3). (D) CDK5-KD and control cells were treated with ibrutinib or vehicle control for 72 hours; proliferation was measured by colorimetric MTS assay (n=9). (E) Ibrutinib resistant and parental cell lines were treated with GFB-12811 or vehicle control for 72 hours; proliferation was measured by colorimetric MTS assay (n=9); CDK5 protein levels were quantified by immunoblotting. (F) Kaplan-Meier survival curve of mice xenografted with JeKo-1 parental and CDK5-overexpressing cells. Two independent experiments, n=10 per group per experiment. Long-rank test, *p<0.05. For immunoblots a representative experiment is shown. Values above each lane represent the mean relative expression (densitometry) normalized to β-actin from three independent experiments. Data are mean ± SEM, *p<0.05, **p<0.01 and ***p<0.001.

Next, we manipulated CDK5 expression in MCL cells to further explore its role in resistance to BTKi. First, we established engineered overexpression of CDK5 in JeKo-1 and Mino cell lines using CRISPR-mediated transcriptional activation (CDK5-OE; Figure 2A). CDK5-OE cells showed increased proliferation compared to control in the presence of ibrutinib (Figure 2B). To generate shRNA-mediated knockdown of CDK5 (CDK5-KD; Figure 2C), we used IR MCL cells which, as noted above, had elevated CDK5 versus their parental counterparts. Loss of CDK5 partially resensitized JeKo-1-IR and Mino-IR cells to ibrutinib (Figure 2D).

Next, we evaluated the effects of a selective CDK5 inhibitor, GFB-12811,^18^ on MCL cell proliferation and survival. Both JeKo-1 and Mino parental cell lines were sensitive to GFB-12811 in a dose dependent manner with IC_50_ of 9.2±2.4 and 11±3.2 respectively (Figure 2E). Meanwhile, IR cell lines demonstrated increased sensitivity to GFB-12811 (Figure 2E). Of note, Mino CDK5-OE cells also exhibited increased susceptibility to GFB-12811 (Supplemental Figure 2).

To determine whether CDK5 upregulation could be a common mechanism of drug resistance not limited to BTKi, we subjected our cells to Bcl-2 inhibitor venetoclax and proteasome inhibitor bortezomib, as both drugs are used in treatment of MCL. Interestingly, Jeko-IR and Mino-IR cells exhibited slightly increased sensitivity to venetoclax, while Mino CDK5-OE was highly sensitive to venetoclax (Supplemental Figure 3A). By contrast, both IR and CDK5-OE cells exhibited bortezomib resistance (Supplemental Figure 3B). These data suggest that CDK5 differentially regulates survival pathways depending on the context.

To elucidate transcriptional differences induced by manipulation of CDK5 expression, we conducted RNA-Seq analysis of CDK5-OE vs. sgNT-JeKo-1 cells, as well as CDK5-KD vs. pLKO.1-JeKo-1-IR cells. We detected expression of 15,145 protein-coding genes across the four tested cell lines. Filtering for genes with padj < 0.05, Fold change > 2, and minimum counts > 10, CDK5-OE led to 41 upregulated and 7 downregulated genes, while CDK5-KD led to 26 upregulated and 161 downregulated genes. Interestingly, we found 9 genes that were upregulated in CDK5-OE and concurrently downregulated in CDK5-KD, including genes known to be involved in carcinogenesis, such as steroid receptor coactivator (Scr kinase) family member proto-oncogen c-Fyn (*FYN)*, niban apoptosis regulator 1 (*NIBAN1;* translational regulator) and tumor necrosis factor receptor superfamily member 4 (*TNFRSF4;* Table 1). Moreover, hallmark pathways ‘TNFα Signaling via NFκB’, ‘Interferon Gamma Response’ and ‘Inflammatory Response’ were upregulated in CDK5-OE cells and downregulated in KD cells, while ‘Oxidative Phosphorylation’, and ‘MYC targets’ were enriched in KD cells and downregulated in CDK5 OE cells (Supplemental Figure 4).

Given the finding of activated pro-survival pathways by CDK5, we sought to further understand how CDK5 might affect lymphomagenesis *in vivo*. NSG mice were xenografted with either CDK5-OE or parental JeKo-1 cell lines as described in the Methods. Mice xenografted with JeKo-1 parental cell line succumbed to disease after 34 days. Meanwhile, mice xenografted with CDK5-OE cell line exhibited a small but significant decrease in survival (median of 30 days; Figure 2F), further confirming the pathogenic role of CDK5.

In summary, CDK5 modulates drug sensitivity and contributes to BTKi resistance and tumor aggressiveness in MCL.

### CDK5 alters proteomic signature in MCL

**Figure 3.**
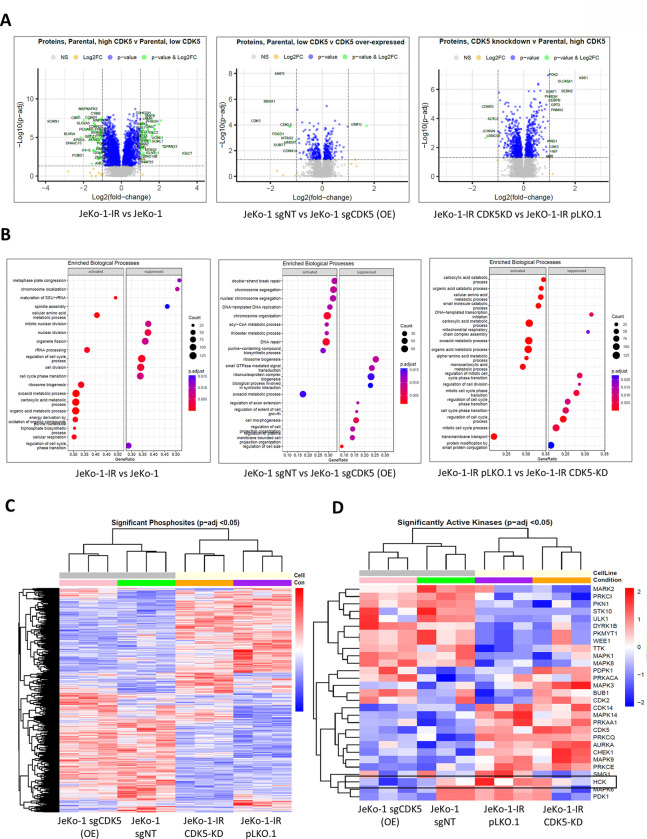
CDK5 regulates proteomic signature in MCL. JeKo-1 parental, IR and with CDK5-manipulated cells (OE or KD) were analyzed by LC-MS as described in the Methods. (A) Volcano plots show differentially abundant protein levels in JeKo-1 models: Left panel: parental vs IR model, middle panel: control vs CDK5 overexpression, right panel: IR vs IR CDK5-KD (n=3). Padj<0.05. (B) Enriched pathway analysis for JeKo-1 vs CDK5 overexpression (left), JeKo-1-IR vs parental (middle) and JeKo-1-IR vs CDK5 knockdown (right). (C) Heat map of significantly altered phosphosites. (D) Heat map of significantly altered kinase activity.

Given that CDK5 is a phospho-kinase we submitted a panel of parental, IR and CDK5-manipulated JeKo-1 cell lines for proteomic analysis. Over 58,000 unique peptides corresponding to over 6,800 unique proteins were detected in each sample, and CDK5 protein abundance matched their genotypes. Comparison between samples revealed over 2,800 differentially expressed proteins in JeKo-1-IR, 395 in JeKo-1 CDK5-OE and 957 in JeKo-1-IR CDK5-KD cells vs. their respective controls (Figure 3A and Supplemental Figure 5A). Pathway enrichment analysis data are shown in Figure 3B. Of the deregulated pathways, ‘G2/M DNA Damage Checkpoint Regulation’ was activated in JeKo-1 cells upon CDK5-OE, while ‘G1/S Checkpoint Regulation’ and ‘ER Stress Pathway’ were downregulated in JeKo-1-IR cells upon CDK5-KD (Figure 3B).

Phosphoproteomic analysis detected over 7,800 unique phosphopeptides for each sample corresponding to 2,640 unique phosphoproteins. Of note, 1,161 phosphosites (569 up, 592 down) were differentially abundant in JeKo-1-IR compared to JeKo-1 parental cells. In CDK5-OE cells, 231 phosphosites (143 up, 88 down) were differentially abundant vs. JeKo-1 parental cells; in CDK5-KD - 375 phosphosites (148 up, 227 down) vs. JeKo-1-IR cells (Figure 3C and Table 2). Kinase activity profiling (IKAP), which estimates kinase activities from substrate phosphorylation levels to help identify active signaling pathways and substrate-kinase interactions, was employed in our dataset to map these phosphosites to kinases according to PhosphoSite Plus dataset and compute each kinase activity (Figure 3D). Twenty-nine kinases were found to be differentially active in at least one comparison between the above pairs. Most of these were kinases involved in cell cycle regulation and DNA damage repair, e.g. WEE1 G2 checkpoint kinase (WEE1), dual specificity protein kinase (TTK) and CDK14. Moreover, most of these kinases play a critical role in lymphomagenesis, among these several mitogen-activated protein kinases (MAPK kinases 1, 3, 6, 8, 9 and 14) and phosphoinositide 3-kinase (PI3K)-related kinases (3-phosphoinositide dependent protein kinase 1 -PDPK1- and slow growth on Galactose and Mannose protein 1 -SGM1-), suggesting their involvement in CDK5-induced ibrutinib resistance.

Additional analysis of kinase activity profiling using kinase substrate enrichment analysis (KSEA) showed a correlation between Src family kinases, particularly with hematopoietic cell kinase (HCK), and CDK5 levels (Figure 3D and Supplemental Figure 5B). These findings were supported by our RNA-Seq analysis which demonstrated an increase in HCK mRNA expression in JeKo-1-IR and CDK5-OE cells, as well as its decreased expression in CDK5-KD cells (Supplemental Figure 6A). These results were further validated by western blot demonstrating that CDK5 protein level in JeKo-1 CDK5-OE cells correlated with increased HCK; similarly, HCK was decreased in JeKo-1-IR CDK5-KD cells (Supplemental Figure 6B). Consistent with the observed HCK protein level increase, cycloheximide chase assay showed a prolonged half-life for HCK in JeKo-1 CDK5-OE cells versus control, together with an increase in one of its downstream targets, the anti-apoptotic Bcl-2 family member Mcl-1 (Supplemental Figure 6C). HCK is known to interact with BTK and is aberrantly expressed in MCL and promotes MCL cell survival and BTKi resistance.^19–21^.

In sum, CDK5 regulates cellular phosphoproteome and kinase activity profile in MCL cells.

### BCR signaling regulates CDK5

**Figure 4.**
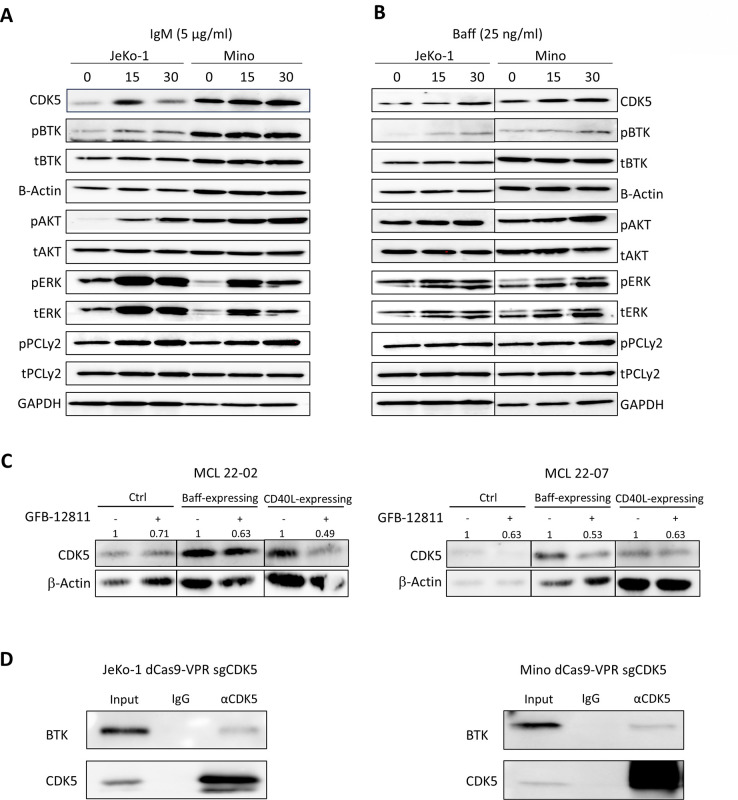
Tumor microenvironment signaling upregulates CDK5. (A-B) Cells were treated with IgM (5 μg/ml) or soluble BAFF ligand (25 ng/ml) as shown and protein lysates were subjected to immunoblotting. A representative blot of 3 independent experiments is shown. (C) Primary MCL cells were cultured in CD40L-, BAFF-expressing stromal versus stroma control for 24 hours, in the presence or absence of 1 μM GFB-12811 and subjected to immunoblotting. Representative experiment is shown. Values above each lane represent the relative expression (densitometry) normalized to β-actin. (D) Protein lysates from JeKo-1 and Mino CDK5 overexpressing cells were subjected to CDK5 immunoprecipitation experiment. A representative blot of 3 independent experiments is shown.

To better understand the regulation of CDK5 in MCL, and given the alterations in oncogenic kinase signaling (MAPKs, PI3K-related kinases and HCK) with CDK5 manipulation, and the dependence of MCL on B-cell receptor (BCR) signaling, we evaluated the levels of CDK5 in JeKo-1 and Mino cell lines after crosslinking with soluble IgM or BAFF ligand, both known to activate BTK.^22^ As expected, stimulation with anti-IgM (5 μg/ml) resulted in increased BTK, PCLγ2, AKT and ERK phosphorylation levels (Figure 4A). Furthermore, we observed a rapid increase in CDK5 protein levels evident at a 15 min timepoint upon IgM-crosslinking which was transient in JeKo-1 cells, but sustained in Mino cells, that appeared to track with BTK phosphorylation (Figure 4A). We have previously shown that B-cell activation factor (BAFF) ligand is capable of inducing BCR signaling in primary lymphoid cells.^22^ To evaluate whether CDK5 is also induced in response to BAFF, we stimulated MCL cells with soluble BAFF. We observed increased BTK phosphorylation albeit of slower onset, which was accompanied by concurrent upregulation of CDK5 in both JeKo-1 and Mino cells (Figure 4B).

Since BCR and BAFF signaling are activated in the tumor microenvironment, we next evaluated CDK5 levels in primary MCL cells cultured in stromal conditions which mimic the lymph node milieu.^23^ Both BAFF-expressing and CD40L-expressing stromal co-cultures induced CDK5 compared with stroma control, and CDK5 levels were further reduced by the CDK5 inhibitor GFB-12811 (Figure 4C).

Given the correlation between BTK phosphorylation and CDK5 expression in BCR-stimulated MCL cells and known BTK scaffolding function,^24^ we conducted immunoprecipitation experiments using JeKo-1 and Mino CDK5-OE cells. In these cells, we observed enrichment for BTK protein in the CDK5 fraction in absence or presence of BCR stimulation, suggesting interaction between these proteins. (Figure 4D and Supplemental Figure 6D). Similar results were observed in Mino-IR cells (Supplemental Figure 6E).

Thus, CDK5 upregulation may be mediated by signals emanating from the MCL microenvironment which are conducted by active BTK.

### CDK5 activates the Unfolded Protein Response (UPR)

**Figure 5.**
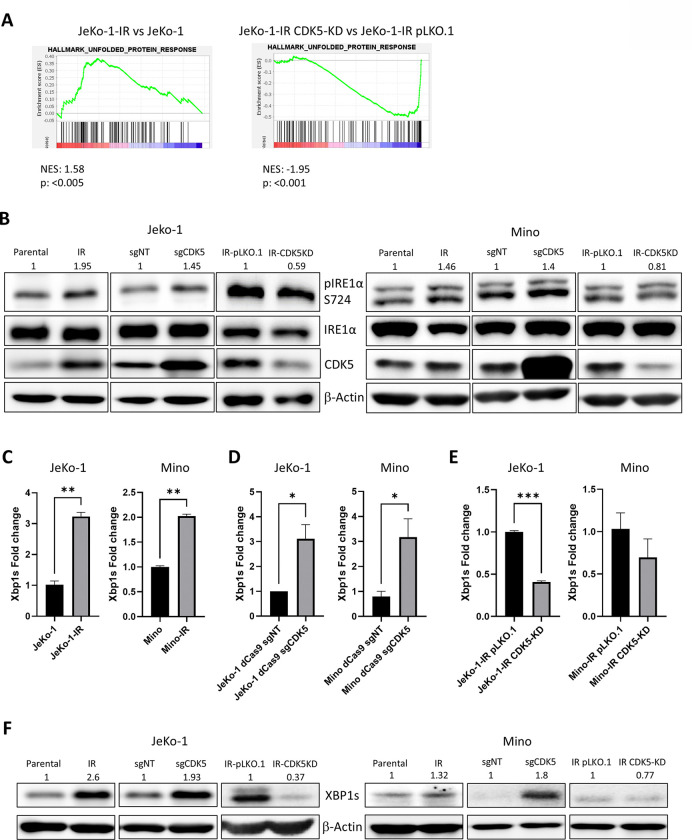
CDK5 upregulates UPR in MCL. (A) GSEA enrichment plots of RNA-Seq results for the UPR pathway in JeKo-1-IR cells (vs. parental – left panel; upon CDK5 KD - right panel). (B) Protein lysates from JeKo-1 and Mino cells with manipulated CDK5 were subjected for immunoblotting. A representative blot of 3 independent experiments is shown. (C-E) *Xbp1s* mRNA levels measured by RT-PCR in MCL cell lines upon CDK5 manipulation. (F) Protein lysates form JeKo-1 and Mino cells with manipulated CDK5 were subjected for immunoblotting. A representative blot of 3 independent experiments is shown. For immunoblots a representative experiment is shown. Values above each lane represent the mean relative expression (densitometry) normalized to β-actin from three independent experiments. Data are mean ± SEM. *p<0.05 and **p<0.01.

Our RNAseq experiments suggested that CDK5 alterations are accompanied by deregulation of the ER stress and the UPR. Work by others suggested that manipulation of CDK5 may deregulate the UPR.^25, 26^ To further analyze whether CDK5 modulates the UPR in context of ibrutinib resistance in MCL, we performed GSEA of RNA-Seq results in a panel of JeKo-1 cell lines. UPR was upregulated in Jeko-IR cells (versus parental) and conversely downregulated in CDK5-KD cells (vs. JeKo-IR transduced with vector control) (MSigDB hallmark gene set collection; Figure 5A). As Xbp1 plays an important role in the most evolutionarily conserved of the three major axes of the adaptive UPR (namely the PERK/ATF4, the IRE1α/Xbp1s, and the ATF6 axes^27^), we further focused on this branch of the UPR pathway. First, we evaluated IRE1α phosphorylation at Serine 724 residue, which controls IRE1α activation state in our manipulated cell lines.^28^ Total IRE1α levels remained constant regardless of CDK5 expression (Figure 5B), in contrast, IRE1α phosphorylation was upregulated in both IR and CDK5-OE cell lines. Meanwhile, CDK5 knockdown led to downregulation of pIREα (Figure 5B). This data suggests that CDK5 activates the IRE1α/Xbp1s axis of the adaptive UPR by phosphorylating IRE1α, causing its activation and inducing Xbp1 mRNA splicing, which then activates downstream components of the UPR.

Next, we checked Xbp1 levels and found that its expression correlated with that of CDK5. Specifically, quantitative PCR using spliced variant-specific primers confirmed that Xbp1s levels were increased in both JeKo-1-IR and Mino-IR cells compared to their parental counterparts (Figure 5C). CDK5-OE led to upregulated Xbp1s mRNA in both cell lines (Figure 5D). By contrast, Xbp1s mRNA levels were downregulated in CDK5-KD cells (Figure 5E). These results were then confirmed by immunoblotting (Figure 5F), suggesting that CDK5 thus regulates Xbp1s.

Activation of the IRE1α/Xbp1 axis regulates a variety of target genes involved in multiple cellular processes including ER stress response, secretory function, lipid metabolism, glucose homeostasis and the inflammatory response^29–32^ and it is also being linked to cancer development and metastasis, regulating among others the pro-survival protein Mcl-1^33, 34^.

**Figure 6.**
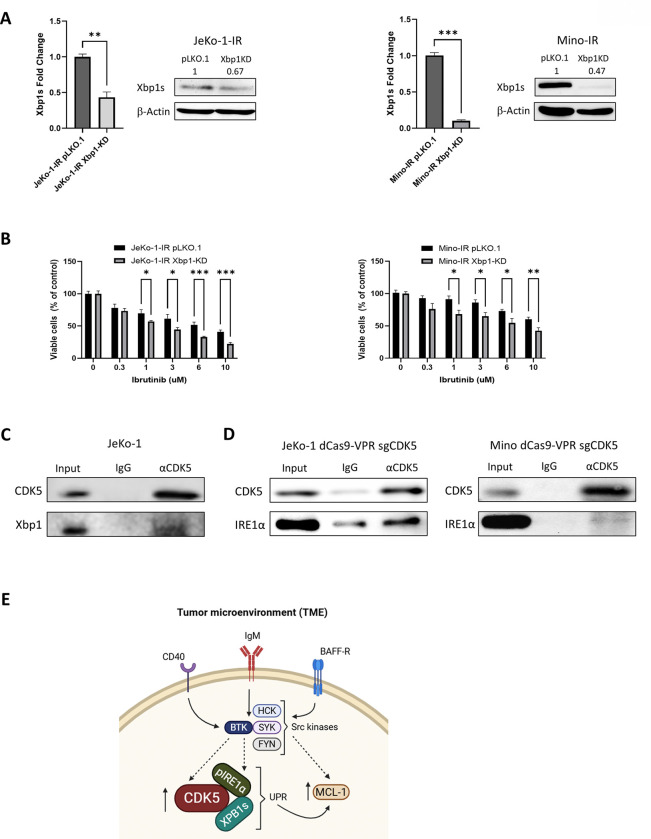
CDK5 interacts with IRE1α and Xbp1s to drive MCL cell survival (A) *Xbp1s* KD was established in IR cells; mRNA and protein levels were quantified by RT-PCR and immunoblotting (n=3). Representative immunoblot is shown. Values above each lane represent the mean relative expression (densitometry) normalized to β-actin from three independent experiments. (B) Xbp1 KD and control cells were treated with ibrutinib or vehicle control for 72 hours. Proliferation was measured by colorimetric MTS assay (n=3). (C- D) Cell lysates from indicated cell lines were subjected to immunoprecipitation with CDK5 antibody and then analyzed by immunoblotting. A representative blot of 3 independent experiments is shown. (E) Schema of CDK5 role in BTKi resistance. Data are mean ± SEM. *p<0.05, **p<0.01 and ***p<0.001.

To further investigate the role of Xbp1s in CDK5-mediated ibrutinib resistance, we conducted genetic knockdown of Xbp1 in JeKo-1-IR and Mino-IR cells (no spliced variant-specific Xpb1s knockdown methodology is available). Quantitative PCR and immunoblot confirmed loss of total Xbp1 in JeKo-1-IR and Mino-IR cells upon shRNA-mediated knockdown (Figure 6A). Cell viability assay assessing proliferation under an ibrutinib concentration gradient indicated that loss of Xbp1 reduced viability of IR cell lines, akin to loss of CDK5 (Figure 6B), suggesting a role for IRE1α/Xpb1s axis and the adaptive UPR in CDK5-mediated ibrutinib resistance in MCL.

Given the correlation between Xbp1 and CDK5 levels in CDK5 manipulated cell lines, we further examined the link between CDK5 and the IRE1α/Xbp1s axis of the adaptive UPR by conducting immunoprecipitation experiments. First, we observed enrichment for XBP1 in the CDK5 pull down fraction in JeKo-1 cells (Figure 6C). Next, due to the relatively low level of endogenous CDK5, we used cells with engineered overexpression of CDK5. Following successful CDK5 pull down in these cells, IRE1α was found to be enriched in the CDK5 fraction in both JeKo and Mino CDK5 OE cells (Figure 6D). This finding indicated an interaction between CDK5 and the IRE1α/Xbp1 axis which to our knowledge has not been previously reported in literature.

In conclusion, CDK5 acts through the IRE1α and Xbp1 to promote UPR and facilitate survival and ibrutinib resistance and presents a potential therapeutic target in MCL (Figure 6E).

## DISCUSSION

Acquired resistance to BTKi represents a major unmet need in MCL. While BTKi therapy has significantly improved patient outcomes, most patients ultimately relapse, underscoring the necessity to identify alternative and complementary therapeutic targets that sustain malignant cell survival once BTK signaling is pharmacologically inhibited.^6^ Increasing evidence suggests that resistance is not solely driven by mutations in BTK or downstream effectors with new/alternate adaptative mechanisms allowing lymphoma cells to tolerate chronic signaling blockade.^11, 35^ In this study, we sought to identify actionable vulnerabilities in BTKi-resistant MCL.

Using well-characterized models of acquired ibrutinib resistance, we demonstrate that BTKi resistance is associated with a distinct transcriptional and proteomic state. RNA-seq analysis of IR MCL cells revealed widespread remodeling of gene expression, including suppression of cell-cycle checkpoint pathways and upregulation of stress-response, inflammatory, metabolic, and oncogenic programs. Similar transcriptional adaptations have been reported in BTKi-resistant CLL and MCL.^36^ Among the most consistently upregulated genes, CDK5 emerged as a candidate of particular interest, showing increased expression at both mRNA and protein levels in IR cells as well as in primary MCL cells resistant to ibrutinib. In agreement with a previous report,^37^ we found that CDK5 was expressed in MCL tumors. Importantly, our analysis of a well-characterized MCL cohort revealed inferior outcomes for patients who expressed high CDK5 mRNA levels,^17^ thus potentially implicating this kinase in general lymphomagenesis and therapeutic resistance in MCL.

CDK5 is not a traditional cell-cycle regulator, and is best known for its role in neuronal development.^12–14^ Yet dysregulated CDK5 activity has been implicated in cancer, where it has been reported to promote tumor growth, metastasis, and therapy resistance in multiple solid tumors.^15, 16^ Conversely, tumor-suppressive roles for CDK5 have also been described, underscoring its context-dependent function.^38, 39^ This duality makes CDK5 an intriguing and understudied candidate in NHL, a disease characterized by profound signaling plasticity.

Functional manipulation of CDK5 further supported its direct involvement as oncogenic driver in the MCL progression, particularly in the context of BTKi resistance. Genetic overexpression of CDK5 promoted survival and proliferation of MCL cells in the presence of ibrutinib, while shRNA-mediated knockdown partially restored drug sensitivity. Pharmacologic inhibition using the selective CDK5 inhibitor GFB-12811 further validated this dependency. These findings are consistent with prior reports linking CDK5 activity to drug tolerance and stress adaptation in cancer cells.^15, 16, 40^ Importantly, CDK5 overexpression correlated with reduced survival in an *in vivo* cell line-derived xenograft model, supporting a role for CDK5 in disease aggressiveness.

Given the central role of B-cell receptor (BCR) signaling in MCL pathogenesis and therapy response, we examined the regulation of CDK5 downstream of BCR activation. Besides the two known activators of CDK5, p35 and p39, little is known about the regulation of CDK5. c-Abl and Fyn phosphorylate CDK5 at Tyr15 and increases its activity in neurons^41, 42^. We show that stimulation with anti-IgM or BAFF induces rapid upregulation of CDK5 in MCL cell lines, in parallel to BTK phosphorylation. Both IgM- and BAFF-mediated signaling have been shown to activate BTK in malignant B cells and are prominent signals in the MCL tumor microenvironment.^22, 43^ Stromal co-culture systems also enhanced CDK5 expression in primary MCL cells, consistent with prior reports demonstrating these models, which mimic the lymph node milieu, promote therapeutic resistance in MCL.^22, 44^ Notably, we identified a previously unreported interaction between CDK5 and BTK, suggesting that CDK5 may physically interact with BCR signaling complex and potentially contribute to recently discovered BTK scaffolding function.^24^

Transcriptomic analysis of CDK5-manipulated MCL cells revealed significant alterations in NF-κB signaling, TNF signaling, inflammatory response, and UPR pathways signaling landscapes, all of which have been implicated in lymphoma survival and therapy resistance.^45, 46^ Besides CDK5, our study identified 9 genes upregulated in CDK5-OE and simultaneously downregulated in CDK5-KD, including FYN (Src family kinase), NIBAN1 (regulator of translation under stress) and TNFRSF4 (TNF receptor superfamily), that have established roles in cytoskeletal dynamics, inflammatory signaling, translation control, and tumor invasiveness and resistance across multiple cancer types.^47–49^ The enrichment of genes with neuronal and cytoskeletal functions further reflects the shared biology between CDK5-driven neurodegenerative and oncogenic processes. Meanwhile, proteomic and phosphoproteomic analyses demonstrated extensive CDK5-dependent remodeling of signaling networks. Although CDK5 itself showed increased activity in IR cells, phosphorylation of canonical CDK5 substrates was not uniformly reduced following CDK5 knockdown. This apparent discrepancy may reflect compensatory activity from either residual CDK5 itself or functionally redundant kinases, a phenomenon commonly observed in kinase-driven resistance states. Our findings also suggest that CDK5 functions as part of a broader adaptive network, rather than acting as a single dominant resistance driver.

Src family kinases, particularly HCK, were identified in both transcriptomic and proteomic datasets. Src kinases are well-established components of BCR signaling and have been implicated in BTKi resistance in B-cell malignancies.^19, 21^ In our model, CDK5 expression correlated with increased HCK stability and elevated Mcl-1 levels, consistent with prior reports linking Src kinases to regulation of anti-apoptotic proteins.^50, 51^

A key mechanistic insight from this study is the identification of UPR activation as a downstream effector of CDK5 signaling. UPR engagement has emerged as a central survival mechanism in cancer cells exposed to oncogenic and therapeutic stress and the IRE1α/Xbp1s axis, which is the most evolutionarily conserved branch of the adaptive UPR has been shown to promote tumor survival, metabolic reprogramming, and resistance to therapy in both hematologic and solid malignancies.^33, 34, 52–55^ Our data demonstrate that CDK5 regulates the IRE1α/Xbp1s axis. We provide evidence of a novel interaction between CDK5 and IRE1α/Xbp1s, which is accompanied by increased phosphorylation of IRE1α at Ser724, a key regulatory site controlling its RNase activity.^28^ Also, we observed a strong correlation between CDK5 expression and Xbp1s levels, and knockdown of Xbp1 partially phenocopied CDK5 loss, reducing viability of IR MCL cells. These findings suggest that CDK5 directly promotes IRE1α activation, leading to enhanced Xbp1 mRNA splicing and sustained UPR signaling revealing the functional importance of this axis.

UPR activation has been linked to resistance to multiple therapeutic agents, including BTKi, BCL-2 inhibitors, and proteasome inhibitors in many cancer types, including diffuse large B-cell lymphoma, multiple myeloma, hepatocellular carcinoma and others. UPR can increase drug detoxification and efflux pump expression, modulate drug targets and upregulate expression of the anti-apoptotic and pro-survival oncogenes.^56–59^ Both BTK and Src kinases have been reported to promote ER stress responses and Mcl-1 stability^23, 50^ and CDK5 has also been implicated in Mcl-1 regulation.^60^ Our data support a model in which CDK5-driven UPR activation contributes to BTKi resistance in MCL and that activation of UPR likely induces therapy tolerance not limited to BTKi in MCL.

In conclusion, our study identifies CDK5 as a central mediator of acquired BTKi resistance in MCL, linking microenvironmental signaling to adaptive UPR activation via the IRE1α/XBP1s axis (Figure 6E). These findings expand current paradigms of BTKi resistance beyond BTK-centric mechanisms and highlight CDK5 and UPR signaling as promising therapeutic targets. Targeting CDK5 or UPR components, alone or in rational combination with BTKi, may offer a novel strategy to prevent and overcome resistance and improve outcomes for patients with MCL.

## Supplementary Material

Supplementary Files

This is a list of supplementary files associated with this preprint. Click to download.

- Table1.xlsx
- Table2.xlsx
- SupplementalMaterial.pdf

Tables

Table 1 and 2 are available in the Supplementary Files section.
